# The Existence of an Intra-Amniotic Microbiome: Assessing a Controversy

**DOI:** 10.3390/biology13110888

**Published:** 2024-10-31

**Authors:** Kumar Uddipto, Julie A. Quinlivan, George L. Mendz

**Affiliations:** 1 School of Medicine, Sydney Program, The University of Notre Dame Australia, 160 Oxford St., Darlinghurst, NSW 2010, Australia; kumar.uddipto@nd.edu.au; 2 Institute for Health Research, The University of Notre Dame Australia, 32 Mouat St., Fremantle, WA 6160, Australia; julie.quinlivan@nd.edu.au

**Keywords:** bacterial taxa, amplicon sequencing, intra-amniotic microbiome, environmental contamination

## Abstract

The existence of intra-amniotic and placental microbiomes during pregnancy has been the source of considerable debate, with research yielding conflicting evidence. This study evaluated investigations into the putative presence of these microbiomes in healthy pregnancies by identifying design and data interpretation issues, particularly concerning contamination in samples with low-density bacterial DNA. Positive findings from diverse populations suggest a consistent presence of microbiota in the intra-amniotic space. Negative conclusions regarding the existence of these prenatal microbiomes emphasise the impact of contamination in the analysis of samples with low-density bacterial DNA. This study concluded that there is no definitive evidence to refute the existence of intra-amniotic microbiomes in healthy pregnancies. Also, it provides suggestions for controlling potential contamination factors in future research on intra-amniotic and placental microbial populations.

## Simple Summary

Investigations during the last few years have shown the central importance of the microbiota of the female genital in pregnancy, both in health and disease. Great advances have been made in understanding the diverse microflora in the vagina and uterus, and significant differences have been found between the microbial densities in both loci, with the intra-uterine populations ordinarily being low mass. The very high sensitivity DNA detection methods employed to identify microbes have the drawback that in studies of low microbial density sites it becomes challenging to differentiate between DNA from contaminants and from resident microorganisms. This problem presents acutely in analyses of the intra-amniotic microbiome, to a degree that many investigators have concluded that there is not such microbiome in healthy pregnancies. In contrast, there are the findings of multiple studies claiming the characterisation of the intra-amniotic microbiome in various female populations. Thus, it became necessary to appraise the evidence in favour and against the existence of this microbiome in healthy pregnancies. This work presents the results of such enquiry and some recommendations to minimise the interference posed by DNA from contaminants.

## 1. Introduction

The placenta, a pivotal organ in pregnancy, and the fetal membranes (the amnion and chorion) play fundamental roles in fetal development and maternal–fetal exchange [1]. The amnion, originating from the epiblast, surrounds the amniotic cavity and serves to cushion and protect the developing fetus [1]. On the maternal side, the chorion interfaces with the maternal decidua, facilitating nutrient exchange through fetal vessels within the villous tree-like structures [1]. The invasion of trophoblastic cells into maternal spiral arteries allows for the perfusion of maternal blood into the intervillous space, ensuring fetal oxygenation and nutrient supply [1]. Understanding these structural aspects of the placenta is crucial as disruptions can lead to various pathologies, including infection and ischemia, which impact on both maternal and fetal health [2]. This study appraised the current debate surrounding the presence of microbiomes in the intra-amniotic space during normal pregnancies. It critically evaluated recent investigations that challenge historical beliefs of intra-amniotic sterility and discussed the implications for clinical practice and future research.

The term microbiome refers to the entire community of microorganisms (viruses, bacteria, fungi, and other microbes) that inhabit a particular environment. The term microbiota refers specifically to the microorganisms themselves. In the context of pregnancy, it is essential to understand the microbiome in the female genital tract and its potential effects on maternal and fetal health, since it provides insights into the mechanisms behind pregnancy-related pathologies such as miscarriage, spontaneous abortion, preterm birth, and placental dysfunction.

The intra-amniotic environment needs to be characterised to understand maternal–fetal interactions. The common causes of early preterm birth involve abnormalities in the intra-amniotic space that result in the inflammation of the chorioamniotic tissues, fetus and uterine structures, leading to the premature shortening and ripening of the cervix; the rupture of the amniotic membranes; placental dysfunction, affecting fetal growth and wellbeing; and direct fetal harm or death [3]. The mother’s genital microbiome has a central role in these interactions [4], and infections directly contribute to adverse pregnancy outcomes such as preterm birth, which is the most common global cause of mortality and morbidity in paediatric medicine [5]. Thus, knowledge of the taxa constituting normal and pathological genital microbiomes is essential in pregnancy care.

In clinical practice, when an infection in the intra-amniotic space is suspected overtly or at a sub-clinical level, the gold-standard diagnostic test is performing an amniocentesis undertaken using sterile techniques, to collect amniotic fluid for culture [6]. A positive culture will confirm a diagnosis of intra-amniotic infection and trigger subsequent management. However, in many cases, cultures are negative, yet the analysis of the placenta following delivery indicates placental evidence of inflammation, including vasculitis and chorioamnionitis. These histological features are independently associated with severe long-term childhood consequences such as cerebral palsy [7]. This approach raises concerns that existing culture-based methods may not detect all intra-amniotic infections; thus, our understanding of the microbiome in this space remains incomplete.

The existence of infection-related intra-amniotic microbiomes is established, but there remains debate about the existence of a microbiome in this space during normal pregnancy. Historically, the human intra-amniotic space was considered sterile, but recent studies employing culture-independent next-generation DNA sequencing techniques have reported the presence of intra-amniotic bacteria in both human- and animal-model healthy pregnancies [8,9]. At variance with these observations, other investigations with intra-amniotic samples have found no evidence of the existence of intra-amniotic microbiome in normal pregnancies [10,11], and support for the sterility of the healthy intra-amniotic space has been sought with examinations of model systems and by re-analyses of published data on this microbiome [12,13].

**Figure 1:**
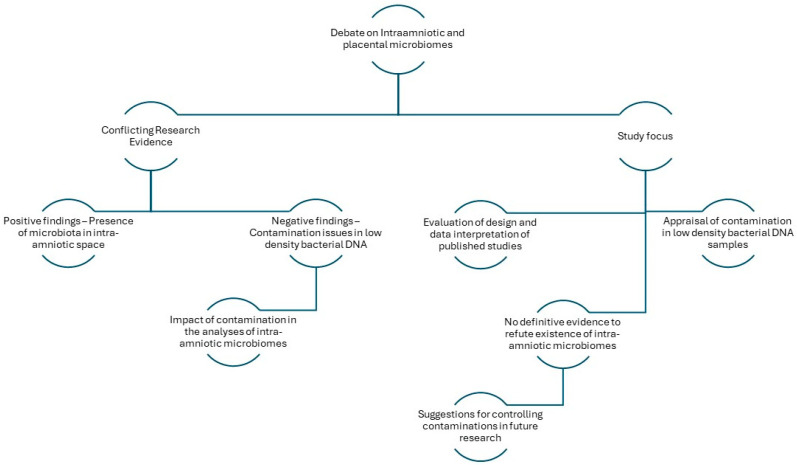
Schematic representation of the architecture of the study.

Conflicting views on the existence of amniotic fluid, placental, or vernix serosa microbiomes are examined by reviewing recent publications that report evidence against or in favour of the presence of these diagram of this work is given in Figure 1.

## 2. Methods

Recent publications were reviewed, with a particular focus on the evidence they provide regarding the presence of microbiota in the intra-amniotic space of healthy pregnancies that resulted in term deliveries in the absence of verifiable infection.

Investigations that concluded the absence of such a microbiome can be distributed into two classes: (i) those that re-analysed data from several previous studies [3,5,6,14,15] and (ii) those that conducted direct investigations into the amniotic fluid, placenta and/or vernix serosa of the fetus and found no evidence of the presence of microbial communities [10,16,17,18]. In this work, both types of studies were appraised by evaluating their robustness, methodological rigour, and interpretation of results.

Numerous investigations have concluded that there are bacterial populations in the intra-amniotic cavity during healthy pregnancies [19,20]. The methods and results are assessed using three studies that employed precautions to minimise DNA contamination and used multiple experimental techniques to verify the microbial sequencing data they obtained.

## 3. Evidence Against the Existence of an Intra-Amniotic Microbiota

Historically, the human intra-amniotic space has been considered sterile, and the presence of microbes in this site was associated with adverse pregnancy outcomes. However, research employing DNA methods of analysis have reported the presence of intra-amniotic microbiota in healthy pregnancies [14]. To resolve conflicting evidence, investigations with intra-amniotic samples and model systems were undertaken to assess the existence of amniotic fluid, placental, or vernix serosa microbiomes in healthy pregnancies.

The DNA present as reagent contaminants has been termed the kitome, and a large number of genera belonging to several phyla have been identified [21]. An investigation employing 16S rDNA and the shotgun metagenomics sequencing of pure *Salmonella bongori* preparations with various DNA extraction kits and ultrapure water showed that, at low bacterial DNA concentrations (obtained by serially diluting the samples), the DNA trace amounts of the extraction kits produced consistent background readings, indicating the need to control for contaminating DNA originating from environmental sources in the analyses of low-biomass samples [21]. The high sensitivity of methods employing DNA for gene surveys to identify bacteria in samples from sites with low-level bacterial biomass must take into consideration the presence of contaminating bacterial DNA in swabs, laboratory dust, extraction kits, PCR reagents, ultrapure water and other commercial chemicals [21]. Several studies demonstrated the presence of technical contamination artifacts arising from reagents or modes of delivery and argued that contaminating microbial DNA in sequencing data from sites of low microbial biomass is likely to be the only source of microbial DNA detected in the intra-amniotic cavity [14,21,22].

An important problem raised in appraising the existence of placental microbiota was the reported diversity in predominant bacterial taxa [14], but these comparisons did not appear to take into consideration maternal racial background [23], living environment, or comorbid medical conditions such as obesity or diabetes [18,24], which influence the composition of bacterial populations in the placenta and other body sites. Another difficulty was the absence or discrepancy between taxa identified by DNA sequencing and those found in results from complementary cultures [14]. To address this issue, it is necessary to understand that laboratory culturing has been unsuccessful for a large number of taxa, and even for some strains of successfully cultured taxa. In addition, under specific growth conditions, some taxa will grow better than others, masking the true abundances in a given niche.

Several studies demonstrate the presence of technical contamination artifacts arising from reagents [12] or modes of delivery [15] and argue that contaminating microbial DNA in sequencing data from sites of low microbial biomass is likely to be the only source of microbial DNA detected in the intra-amniotic cavity. This reasoning requires examination.

In environments where it is known there exists low microbial biomass, there could be problems distinguishing between taxa belonging to this biomass and those added by technical contamination. The difficulty would hinge on detection sensitivity and would explain the observed variations between controls and biological samples. On the other hand, to ascertain the existence of a low biomass, the contamination argument cannot be used. In the investigation of a specific niche, if differences between controls and biological samples were observed, an explanation must be provided for the various alpha diversities measured in controls and in samples, as well as the beta diversity differences between controls and samples. The differences cannot be ascribed to limits in DNA detection sensitivity.

### 3.1. Re-Analyses of Published 16S rRNA Sequencing Data

To investigate whether a microbiota inhabits the intra-amniotic space and the placenta in healthy pregnancies, two re-analyses of recently published data were examined.

A critical review of microbiota datasets was conducted by Panzer et al. [14], re-analysing published data employing the DADA2 pipeline. In this study, the selection of samples used to examine *Lactobacillus* amplicon sequence variants (ASVs) in placental samples is unclear, and the conclusion that their source was contaminants is at variance with the studies quoted that conclude the maternal side of the placenta had a high predominance of *Lactobacillus* [14]. In eleven of the datasets, the beta diversity of the ASVs of placental samples was compared to those of technical controls employing principal component analysis (PCA), and it was concluded that both sets of ASVs partially clustered to various degrees; thus, contamination would be the origin of the DNA detected in placental samples. However, the data shown in Figure 3 of the study did not support this conclusion; most placental ASVs do not overlap with the ASVs from reagents or the environment.

This work also examined the top relative abundance ASVs in six studies that employed primers targeting the V4 region of the 16S rRNA genes, by comparing PCA plots (Figure 5) and heatmaps (Figure 6) of the beta diversity of placental and technical control samples and their relative abundances. It was concluded that these “prominent placental ASV were likely derived from background contamination captured by the technical control samples” [14], but this conclusion is not supported by the data shown in these figures. In the PCA plot shown in Figure 5a, multiple placental ASVs do not overlap with those of the technical controls; in particular, the beta diversities various ASVs of *Lactobacillus* in placental and technical controls are clearly different. In addition, in the heatmaps of Figure 6, most of the reported taxa ASVs detected in placental samples are either absent or significantly smaller in technical control samples.

This comparative work highlighted significant differences in the degree and types of contaminant DNA between studies and the need to control this contamination, but it did not provide evidence against the presence of a placental microbiome in human pregnancies at term.

Re-analysing published 16S rRNA gene datasets, another study questioned whether the intra-amniotic environment is stably colonised by microbial communities in healthy pregnancies given the low-biomass nature of the proposed microbiome, and the high sensitivity of the DNA sequencing meant detected ASVs were more likely from various types of contamination [15]. Their re-analyses of microbial sequencing profiles from the three datasets shown in Figure 1 yielded very significant differences in the taxa relative abundances between rectal swabs of an infant delivered by C-section and intestinal swabs of fetuses delivered vaginally.

The validity of these comparisons is questionable considering the strong dissimilarities between the sequences found in rectal and intestinal swabs and the similarities in relative abundances observed between the two studies analysing intestinal swabs. Further, the fetal meconium is not passed unless a fetus is distressed and may not ever be representative of an intra-amniotic microbiome of a normal pregnancy, as there is traditionally considered to be no passage of meconium into the amniotic fluid in healthy pregnancies, unlike the urinary system, where the fetus engages in the constant swallowing of amniotic fluid and voiding into this space [25,26]. Thus, one might not expect any commonality in newborn rectal and intra-amniotic microbiomes in a healthy pregnancy. Meconium should not be found in the amniotic fluid of healthy pregnancies, given it is a clinical marker of intrauterine fetal distress if detected at delivery, unless an infant is post-term on the date of delivery [25,26]. In the studies analysed, the samples were not from post-date pregnancies. The selection of the newborn rectal sample was therefore an inappropriate comparison reference.

There were several-fold increases of *Lactobacillus*, *Staphyloccocus* and *Afipia* relative abundances in placental swabs compared to technical controls from reagents and the environmental samples that argue against contamination as a sole explanation [15].

It was also asserted that the results of sequencing meconium samples from extremely premature infants showed many reagent contaminants as well as genuine signals—at variance with the results from intra-amniotic fluid samples. The data from meconium samples obtained from neonates delivered by C-section show that about 50% of the total sequence reads contained less than 1% DNA from technical controls, and the DNA of reads considered to be genuinely sample-associated yielded six different most-abundant taxa in these samples [15]. The latter results were dismissed as evidence of an intra-amniotic microbiome by attributing their origin to the skin of the mother, although “there have been few attempts to track species and strains to confirm fetal origin” [15]. Moreover, even if it were considered reasonable to compare the bacterial DNA in meconium samples with that in amniotic fluid, assuming some aspiration of the latter by the fetus, it is less reasonable to assume that the amniotic fluid will include all types of bacteria present in the intra-amniotic space by ignoring the differences between planktonic and biofilm-forming taxa that may preferentially locate within the amniotic fluid or the placenta and the skin of the fetus, respectively. Importantly, in meconium samples, there was no DNA from *Atopobium*, *Gardnerella*, *Lactobacillus*, *Prevotella* or other taxa found frequently in vaginal and intra-amniotic samples, raising doubts about extending the conclusions from meconium microbiota investigations to intra-amniotic microbiome studies.

The discussion of batch effects assumes that the relative abundances of specific taxa have to be the same in all batches [15]. This assumption may be correct for technical control samples in the absence of any other DNA, as shown in the report, because samples would be near-identical, e.g., buffers, air swabs. However, the assumption is not necessarily correct for biological samples, where the collection itself includes a degree of heterogeneity, even in high-microbial-biomass samples such as vaginal ones. In addition, the evident higher relative abundances of *Gardnerella*, *Lactobacillus* and *Prevotella* and others in the intestinal samples indicate that the corresponding ASVs cannot all have their source in the technical controls. Thus, the presumed batch effects remain to be demonstrated in this study [15].

A problem with the conclusions of this work is that an explanation must be provided for the various alpha diversities measured in controls and in samples, as well as the beta diversity differences between controls and samples. The response sensitivity of the 16S rRNA sequencing is such that only contaminant DNA is detected in some samples, and this does not explain the variations in diversity [15].

This study commonly reaches conclusions about the origin of taxa by attributions rather than by observations. It includes long discussions about implications of the presence of bacteria in the intra-amniotic cavity that would require a revision of many perspectives of reproductive biology, microbial ecology, bioinformatics, immunology, and clinical microbiology. This is correct, but required changes in accepted perspectives cannot be taken as evidence of the absence of an intra-amniotic microbiome.

### 3.2. Investigations That Did Not Find Evidence of an Intra-Amniotic Microbiome

A study of 42 amniotic fluid samples employed 16 positive control and 12 negative control samples comprising sterile saline, buffers, reagents and DNA-free water blanks. The methods used to detect the presence of microorganisms were cultivation, qPCR and 16S rRNA gene sequencing [18]. However, the collected amniotic fluid was centrifuged at 1300× *g*, and the samples employed in the various experiments were aliquots of supernatants; there is no information about the pellets. Centrifugation is often employed to disrupt the cell membrane and release the cytosol content, including nucleic acids. This process is known to compromise the growth rate and viability of planktonic bacteria in liquid cultures [27], and overall, it could dilute the bacterial density in samples by precipitating microbial cells. Both effects render uncertain the reported negative results of the cultivation bacteria in liquid media.

Further, samples of amniotic fluid were collected during the Caesarean section, and it is standard practice to administer a prophylactic intravenous antibiotic at this time that may interfere with subsequent cultures, especially if intra-amniotic samples were collected immediately after antibiotic administration. This detail is omitted in the study methods, so the timing of sample collection relative to antibiotic administration is unknown.

The absence of significant differences in gene copy numbers and richness between in the amniotic fluid samples and sampling controls supported the conclusion that there were no microorganisms in the mid-trimester amniotic fluid of healthy pregnancies [19]. In these measurements, the DNA extracted from samples was diluted 1:20 to perform qPCR amplifications, and further 1:5 and 1:20 dilutions were performed to amplify and sequence it. These levels of dilution will increase the DNA concentrations in the reagents relative to that in the samples. Notwithstanding this methodological approach, this study shows differences between the gene copy numbers in amniotic fluid samples and in the extraction reagents and blank controls; also, the notable difference between the tightly clustered data points of the controls and the approximately one-order-of-magnitude spread of the data points of the amniotic samples indicates variations between the latter samples, which are not observed in the sampling controls [19]. The data from principal component analyses manifest marked differences in the spread of bacterial richness between amniotic fluid samples and sampling controls, also showing that most taxa present in the biological samples are different from the ones in the controls [19]. Thus, the data presented in this study do not provide sufficient evidence to conclude that there is no microbiota in the amniotic fluid of healthy pregnancies.

An investigation of 76 pregnancies that reached term did not find evidence to support the existence of a placental microbiome, understood as a stable bacterial population in a body site [10]. The participants included women older than 18 years who had full-term pregnancies—50 who underwent elective Caesarean delivery and 26 who underwent vaginal delivery. Samples were collected from the vernix caseosa of the infant’s armpit or groin, the amniotic fluid, and the maternal, medial and fetal layers of the placenta. Timing in relation to the surgical administration of antibiotics is not reported.

Some samples yielded live colonies of bacteria, including those from Caesarean delivery placentas, but owing to most being bacteria commonly found on the skin, it was surmised that their origin was contamination during surgery. Surprisingly, it was not discussed that in many samples, higher numbers of colony-forming units were counted in Caesarean-delivered placental samples than in vaginal-delivered ones, and we observed significantly different relative taxa abundances in both modes of delivery. It could be expected that a greater diversity and relative abundance of skin taxa would be present in samples from vaginally delivered infants, but in a number of instances, the opposite was observed [10]. Hence, the inference that the taxa identified were contaminants from the surgical procedures is not supported by the evidence. Moreover, Caesarean-section surgery is undertaken using aseptic techniques with surgeon fully scrubbed, gloved and gowned. The surgical field is prepared with topical agents to prevent infection and surgery performed using aseptic techniques. In clinical medicine, when swabs are collected from the placenta in a setting of suspected infection and are positive, they are considered a true infection and not a contamination.

Taxa detected using 16S rRNA amplicons were found in placental samples from Caesarean deliveries, amniotic fluid and vernix caseosa samples, and it was concluded that “bacterial signals detected from placental tissue were not distinguishable from background signals, except in some individual cases”. A similar interpretation was applied to samples from the other sites. However, different taxa relative abundances are shown in the data from the placental samples obtained from the maternal, middle and fetal layers, with larger taxa numbers and abundances in the maternal-side placental samples. In fact, the study states that the gene counts on these samples reflect the presence of bacteria at this site [10].

Growth-inhibition experiments were conducted with *E. coli* cultures in the presence of placental samples, and the data show the inhibition of bacterial growth, but it is an unwarranted extrapolation to infer that this would apply to all bacteria and thus that it reveals antimicrobial activity in the placenta [10]. More likely, it may relate to concurrently administered intravenous antibiotics at the time of sample collection and that these antibiotics may be the source of antimicrobial activity. Again, timing of sample collection and results are not stated in the methods. From a clinical medicine perspective, the intravenous antibiotics are routinely administered before the delivery of the baby to achieve peak prophylactic surgical-site infection efficacy, and it is unlikely that attending clinicians would have compromised this for research purposes.

The overall conclusion that there is no evidence of the existence of a placental microbiome is not supported by the data presented, even when the definition of the microbiome is that of a stable bacterial population at a given site. Moreover, the study demonstrates differences in the microbiota in three layers of the placenta. With this heterogeneity in mind and considering that placental samples were all collected from a narrow region about 4 cm from the cord insertion site, it does not seem reasonable to extrapolate the results to the entire organ [10].

Other recent studies discuss the presence of contaminating bacterial DNA in intra-amniotic samples [28,29,30], but they do not provide conclusive evidence of the absence of microbiota at this locum.

## 4. Evidence Supporting the Existence of an Intra-Amniotic Microbiota

The presence of bacteria in the uterus [31,32] and endometrium [33] has been described, but the question is whether there is a microbiota in the intra-amniotic space during heathy pregnancies.

The microbiota at the basal plate, placental villi, and fetal membranes were investigated in 57 American women with term pregnancies [34]. It employed sequencing multiple variable regions of the bacterial 16S rRNA gene to assess alpha and beta diversity, as well as the absolute abundance of this gene at each location. The most highly prevalent operational taxonomic units (OTUs) in each niche were identified, and OTUs detected in negative blank and water controls were excluded. The work found that bacterial populations exhibit distinct profiles at each location. The finding that distinct bacterial taxa dominate in the three placental niches was validated by quantitative PCR analyses. The study concluded that “placental microbiota and placental microbiome studies should consider regional differences, which may affect maternal, fetal, and/or neonatal health and physiology” [34].

The placental microbiota of 61 Chinese participants was investigated using 16S rRNA gene sequencing in a cohort with 10 women with gestational diabetes mellitus (GDM), 19 women with premature rupture of membranes (PROM), 11 women with other pregnancy complications, and 21 women delivering at term without adverse complications [35]. In addition to the 61 placental samples, there were 10 environmental control samples. The work identified 871 OTUs in the placental samples and 503 OTUs in controls, including scissors, sheets, cotton swabs, etc., as well as those from exogenous marine bacterial DNA, with 300 OTUs common to both sample sets. Principal component analyses of the 571 OTUs unique to the placental samples showed that the microbiota of women with healthy pregnancies is significantly different from those with GDM and PROM, and there was no significant difference in placental microbiota composition between the latter two groups of women. The finding that low amounts of microbiota in human placenta that varied between women with healthy pregnancies and those with GDM and PROM prompted the need to take into account potential environmental contamination, leading to the standardisation of operating specifications and procedures to study low-mass microbiota [35].

To investigate the role of placental dysbiosis in pregnancies with late fetal growth restriction (FGR), a study with 36 Caucasian women compared the proteinograms of 18 women with normal pregnancies and 18 women with late FGR diagnosed after 32 weeks of pregnancy [36]. Liquid chromatography–mass spectrometry was employed to identify bacterial proteins present in the placental biomasses. In the normal placentas of the control group, 628 proteins were found, and 145 proteins were found in the placentas of the FGD group, with 52 proteins common to both groups. Based on proteomic data, in the control group, the statistically more frequently taxa were *Flavobacterial bacterium*, *Aureimonas* spp. and *Bacillus cereus*. It was hypothesised that the presence of numerous bacterial proteins in normal placentas may indicate a protective role of the corresponding bacteria, and the bacterial proteins detected only from the placentas of FGR pregnancies suggested a potentially pathogenic nature of the bacteria expressing them [36].

Multiple recent studies that employed a variety of methods and detailed various precautions taken to minimise the effects of potential sources of DNA contamination have reported the presence of a microbiota in healthy pregnancies [37,38,39,40,41,42].

## 5. Discussion

There is agreement that contamination introduces important problems into the analyses of microbiota from low-biomass samples, but there is no consensus on whether there is a low-bacterial-density intra-amniotic microbiome in healthy pregnancies. Several investigations demonstrated that contamination is the origin of some taxa identified in these microbiomes. Nonetheless, the claims of a few studies extrapolating these findings to negate the existence of such microbiota remain to be convincingly proven.

Variations in the selection of bioinformatics tools for analyses can significantly influence interpretations of microbial population data in specific anatomical loci. Differences in sample collection methods, DNA extraction techniques, sequencing technologies, and data analysis approaches can introduce variability in the results, which affects the accuracy and reliability of microbial community profiles. In turn, this variability could lead to differing conclusions about the composition, diversity, and functional potential of the microbiome in a given locus. Therefore, it is crucial to standardise methodologies and employ robust bioinformatics tools to ensure consistent and accurate interpretations of microbial populations.

No definitive evidence has resulted from re-analyses of published amniotic fluid and placenta microbiota data, or from studies of intra-amniotic samples concluding to have found only contaminant taxa. On the other hand, a growing body of investigations that applied stringent measures to minimise the potential effects of contaminants has consistently reported the presence of bacterial taxa during healthy pregnancies within this assumed sterile environment. These findings are not universally accepted but provide a compelling reason that warrants further investigations.

## 6. Conclusions

The persistence of the debate on the existence of an intra-amniotic microbiome indicates the need for more investigations that apply measures specifically designed to minimise technical contamination. This will serve to enhance the reliability and validity of the results of such studies. Collaborative efforts encompassing standardised protocols and analytical approaches will help unravel the complexities of microbial populations within the intra-amniotic space.

To support this effort, a set of guidelines have been devised that would minimise the impact of technical contamination. A summary of these recommendations is as follows:

(i) To employ qualified persons and appropriate sterile techniques to collect swabs; extract and amplify the DNA; and prepare samples for sequencing. To secure uncontaminated intra-amniotic samples for a research study, the same principles need to be followed as in the collection for clinical diagnostic purposes. Samples need to be collected by a trained specialist in obstetric medicine familiar with sterile operating techniques. The collection should be performed under surgical aseptic techniques, and the samples should be stored in appropriately sterile environments. In the case of placental samples, it needs to be specified whether they were collected from the surface of the placenta or from the space between the placental membranes, as well as what precautions were taken to minimise contamination. At least duplicate samples need to be acquired to check for contamination. The timing of sample collection in relation to the administration of any surgical antibiotics should also be specified.
(ii) To include positive and negative controls in the sequencing of biological samples. These will reveal potential contamination incurred in the DNA preparation for sequencing arising from reagents, laboratory environment and instruments. To avoid batch effects, the sequencing of dual samples should be spaced, and controls should be intercalated.
(iii) To use various experimental techniques to verify the presence of microbiota in the intra-amniotic cavity. A standard procedure to discard taxa with less than 1% relative abundance could result in a loss of information but will contribute to minimising the potential contaminant’s contribution to the data.
(iv) To conduct analyses of sequence data with bioinformatics tools that help discriminate between contaminants and samples. The development of analytical pipelines and tools will improve the ability to discriminate between taxa from contaminants and from low-biomass sites.

The adoption of these measures in future studies will assist, as they will void the methodological concerns reported to date.
